# The influence of liver and pancreas surgery on the thyroid function

**DOI:** 10.1186/1756-6614-5-21

**Published:** 2012-12-21

**Authors:** Katarzyna Wojciechowska-Durczynska, Arkadiusz Zygmunt, Adam Durczynski, Janusz Strzelczyk, Andrzej Lewinski

**Affiliations:** 1 Department of Endocrinology and Metabolic Disease, Medical University of Lodz, Polish Mother’s Memorial Hospital – Research Institute, Lodz, Poland; 2 Department of General and Transplant Surgery, Medical University of Lodz, Norbert Barlicki Teaching Hospital, Lodz, Poland

**Keywords:** Abdominal surgery, Thyroid function

## Abstract

**Background:**

Nowadays, the increasing number of oncologic patients with liver or pancreatic tumours are subjected to surgical treatment, as it can provide a long-term survival or sometimes cure. As a result, numerous new clinical questions regarding metabolic disturbances in these patients have been arisen. Among others, the impact of the pancreas and liver surgery extent in relation to the thyroid function remains to be elucidated.

**Materials and methods:**

The study comprised 51 patients (25 men and 26 women, mean age ± SD 61.6 ± 10.4 yrs, mean ± SD) with pancreatic or liver tumours, qualified for abdominal operation. Serum levels of FT3, FT4 and TSH were measured on the day before (time “0”) and on the 1^st^, 3^rd^ and 5^th^ day after surgery in two (2) subgroups reflecting the extent of surgery: twenty seven (27) patients (14 men and 13 women, mean age ± SD 61.5 ± 11.8 yrs) after major surgery (Whipple’s surgery, right and left hemihepatectomy, segmentectomy of the liver, distal pancreatectomy, total duodenopancreatectomy) and twenty four (24) patients (11 men and 13 women, mean age ± SD 61.8 ± 8.9 yrs) after minor, palliative surgery (exploratory laparotomy, gastroenterostomy, triple by-pass, liver tumour embolization, hepaticojejunostomy). Additionally, the obtained results were analyzed in relation to the type of the disease (pancreatic surgery vs liver surgery).

**Results:**

Mean serum FT3 level decreased significantly during the study in major and minor surgery subgroups (p<0.001, in both) in comparison to the baseline values, accompanied by stable serum concentrations of TSH (NS) and FT4 (NS). The above decreasing tendency in FT3 concentrations was similar in both subgroups (NS), the same as were unchanged levels of TSH (NS) and FT4 (NS). Mean FT4 concentration on the 3^rd^ and 5^th^ day after major surgery was lower in pancreatic tumour patients in comparison to liver tumour patients (p=0.002, p=0.032, respectively). Similarly, mean FT3 concentration on the 3^rd^ day in minor surgery subgroup was lower in pancreatic tumour patients in comparison to liver tumour patients (p=0.015).

**Discussion:**

Our findings have confirmed essential reduction of FT3 values after abdominal surgery, independently of surgery extent. Additionally, pancreatic tumour patients are more likely to have lower FT3 and FT4 levels after surgery when compared to liver tumour patients.

## Background

The condition called “low 3,5,3^′^-triiodothyronine (T3) syndrome” or “nonthyroidal illness syndrome (NTI)” or “euthyroid sick syndrome” is characterized by low circulating triiodothyronine (T3) levels without an initial rise in thyroid stimulating hormone (TSH) in critically ill patients [1,2]. Additionally, if the severity of illness increases, there is a decrease in both serum T3 and thyroxine (T4) [3]. Abnormalities in thyroid hormone metabolism reflect the severity of illness or trauma what speaks in favor of the concept that these syndromes can be used for prognostic purposes [2,4,5].

It is well known that the surgery causes complex metabolic alterations and affects circulating thyroid hormone concentrations, as well [6,7]. However, the potential impact of various surgical procedures on the thyroid function remains not sufficiently clear.

Nowadays, the increasing number of oncologic patients with liver or pancreatic tumours are subjected to surgical treatment, as it can provide a long-term survival or sometimes cure. As a result, many new clinical questions regarding metabolic disturbances in these patients have been arisen.

In order to evaluate the effect of the pancreas and liver surgery extent on the thyroid function, we measured free T3 (FT3), free T4 (FT4) and thyrotropin (TSH) concentrations before and right after major and minor abdominal surgery, looking for possible changes. Purposefully, we have not decided to perform more complicated thyroid function tests such as reverse T3 and T3 resin uptake, in hope of finding correlation between the extent of liver and pancreas surgery and thyroid hormone levels alterations measured by tests routinely available in all laboratories.

## Materials and methods

The study was approved by the Ethics Committee of Medical University of Lodz, Poland. We investigated serum samples collected from 51 patients (25 men and 26 women, mean age ± SD 61.6 ± 10.4 yrs) with pancreas or liver tumours, qualified for abdominal operation. The patients recruited to the study did not have any known or clinically suspected thyroid dysfunction. Additional exclusion criterion was treatment with thyroid hormones or antithyroid drugs. The patients were divided into two (2) subgroups reflecting the extent of surgery. Twenty seven (27) patients (14 men and 13 women, mean age ± SD 61.5 ± 11.8 yrs) with resectable tumours underwent major surgery: Whipple’s surgery (n=13), right and left hemihepatectomy (n=6), segmentectomy of the liver (n=4), distal pancreatectomy (n=3) or total duodenopancreatectomy (n=1). Twenty four (24) patients (11 men and 13 women, mean age ± SD 61.8 ± 8.9 yrs) with unresectable tumours underwent minor, palliative surgery: exploratory laparotomy (n=9), gastroenterostomy in unresectable pancreatic tumours (n=6), triple by-pass (n=4), liver tumour embolization (n=4) and hepaticojejunostomy (n=1).

Serum concentrations of FT3, FT4 and TSH were measured on the day before surgery (time “0”), and also on the 1^st^, 3^rd^ and 5^th^ day after surgery. Additionally, the obtained results were analyzed in relation to the type of the disease (pancreatic surgery vs liver surgery).

We have used a one way repeated measures analysis of variance for comparison of mean concentrations of TSH, FT3 and FT4 in four (4) measurements. We have employed the Fisher LSD method for comparison of FT3 concentrations in each subsequent measurement in relation to blood collection time point, before and after surgery, respectively. In order to compare the differences in TSH serum concentrations between patients who underwent minor and major abdominal surgery, we have applied the parametric *t*-test and non-parametric Mann-Whitney’s and Kruskal-Wallis’ tests. The *t*-test, equal variance test and Holm-Sidak method have been performed for demonstration differences in FT3 and FT4 concentrations between aforementioned groups. Furthermore, Mann-Whitney’s and equal variance tests have been carried out to compare TSH, FT3 and FT4 after liver and pancreas surgery in two (2) previously analyzed subgroups. In all analyses, statistical significance has been considered achieved at a value of p<0.05.

All statistical calculations and graphs have been performed using SigmaPlot version 12.0 (Systat Software Inc., San Jose, CA, USA).

## Results

**Figure 1:**
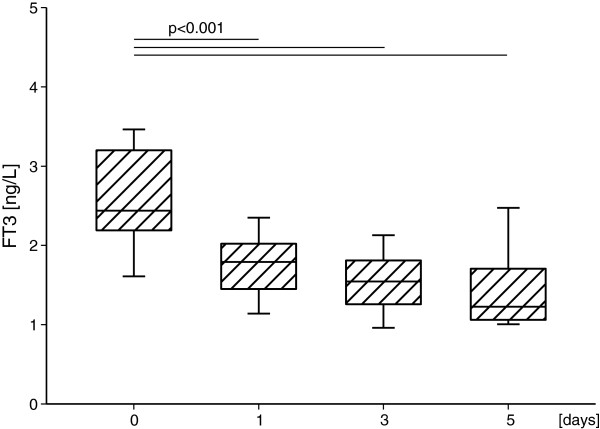
**FT3 serum concentration in the major surgery subgroup on the subsequent days.** Upper and lower limits of boxes are 75 and 25 percentile, respectively. Horizontal line in boxes represents mean value. Whiskers illustrate SD.

**Figure 2:**
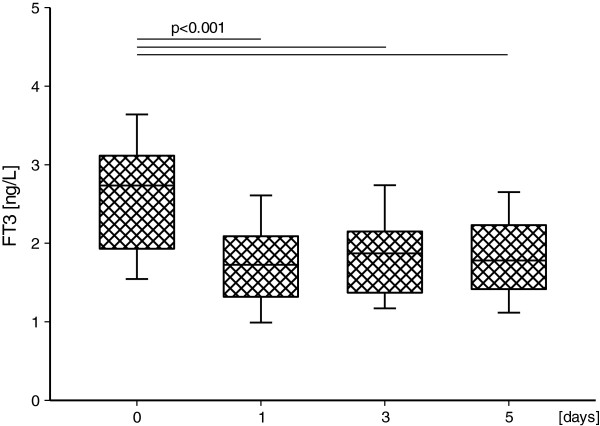
**FT3 serum concentration in the minor surgery subgroup on the subsequent days.** Upper and lower limits of boxes are 75 and 25 percentile, respectively. Horizontal line in boxes represents mean value. Whiskers illustrate SD.

Mean values of FT3, FT4 and TSH on the following days are presented in Table 1 and Table 2. Mean serum FT3 level decreased significantly during the study in major (Figure 1) and minor (Figure 2) surgery subgroups in comparison to time “0” values (p<0.001, in each case), accompanied by unchanged serum level of TSH (NS) and FT4 (NS). The above decreasing tendency in FT3 concentrations was similar in both subgroups (NS), the same as were unchanged levels of TSH (NS) and FT4 (NS).

**Table 1:** Mean values of FT3, FT4 and TSH concentrations on the subsequent days in major and minor surgery subgroups

		**Major surgery subgroup**	**Minor surgery subgroup**
N		27	24
Men		14	11
Women		13	13
Age		61.5 ± 11.8	61.8 ± 8.9
FT3 [pg/mL] N: 1.71-3.71	0	2.614	2.662
	1	1.733	1.727
	3	1.561	1.798
	5	1.440	1.825
FT4 [ng/dL] N: 0.7-1.48	0	1.157	1.214
	1	1.141	1.253
	3	1.155	1.172
	5	1.075	1.148
TSH [mIU/L] N: 0.35-4.94	0	1.219	1.245
	1	1.164	1.157
	3	1.166	1.513
	5	1.376	1.689

**Table 2:** Mean values of FT3, FT4 and TSH concentrations on the subsequent days in pancreatic and liver tumor patients’ subgroups

		**Major surgery subgroup**		**Minor surgery subgroup**	
		**Pancreatic tumor patients**	**Liver tumor patients**	**Pancreatic tumor patients**	**Liver tumor patients**
N		17	10	17	7
FT3 [pg/mL] N: 1.71-3.71	0	2.587	2.659	2.739	2.473
	1	1.819	1.585	1.718	1.749
	3	1.577	1.535	1.665	2.295
	5	1.358	1.605	1.893	1.0
FT4 [ng/dL] N: 0.7-1.48	0	1.116	1.226	1.222	1.194
	1	1.131	1.16	1.218	1.337
	3	1.082	1,272	1.147	1.265
	5	1.017	1.191	1.176	0.81
TSH [mIU/L] N: 0.35-4.94	0	1.258	1.152	1.292	1.129
	1	1.277	0.971	1.069	1.369
	3	1.080	1.302	1.458	1.719
	5	1.445	1.239	1.592	2.858

In the group of patients with pancreatic tumours who underwent major surgery it was found that FT4 concentration was lower on the 3^rd^ day (1.082 ± 0.144) than in subjects with liver tumours (mean ± SD 1.272 ± 0.108) (p=0.002). Similar differences were observed on the 5^th^ day (median 1.035 vs 1.185) (p=0.032).

In the group of subjects after the minor operation, FT3 concentration on the 3^rd^ day after the surgery was lower in the patients with pancreatic disease (1.629 ± 0.458) than in the patients with liver tumours (mean ± SD 2.295 ± 0.412) (p=0.015). Other differences were not statistically significant.

## Discussion

Despite the description of low T3 syndrome above 40 years ago, its pathogenesis has not been completely understood. Various parameters have been considered to play role in this syndrome, including inhibitors of T3 or T4 binding to proteins, decreased deiodinase 1 (D1), and deiodinase 2 (D2) activities and increased deiodinase 3 (D3) activity, as well as are tumour necrosis factor α (TNFα), interleukin 6 (IL-6) [8]. In addition, one of the dilemmas is the significance of low T3 syndrome in critically ill patients. Some researchers demonstrated a protective function of that phenomenon [9], while others reported rather as either an adaptive process in the condition of reduced energy supplies or a maladaptive one, resulting in tissues impairment [10].

When discussing the low T3 syndromes, it should be mentioned that this phenomenon occurs immediately after operation due to stress [7]. Disturbances of thyroid function can be observed within the first hours after skin incision [8]. Furthermore, patients with low T3 level were older than those with normal T3 level and in case of critically ill patients, low T3 syndrome was much more frequently found in the non-survivors than in survivors; consequently, the case fatality rate (CFR) was much higher in the group of patients with low T3 level than in patients with normal T3 [11].

Our findings have confirmed reduction of FT3 values on 1^st^, 3^rd^, 5^th^ day after surgery in both studied groups, though the FT3 values decreased below the reference range only in 3^rd^ and 5^th^ day after major surgery. Thus, our observations are in compliance with previous studies that proved the presence of low T3 syndrome in patients after minimally invasive laparoscopic cholecystectomy [7] and after major cardiopulmonary bypass surgery [12,13]. Additionally, just as expected, patients with low T3 concentrations before surgery demonstrated postoperatively a more severe degree of low T3 syndrome [12].

The comparable results of thyroid function tests in two analyzed subgroups provide some evidence that the extent of abdominal surgery does not impact significantly on the thyroid condition. This part of our results is in agreement with prior study [14] in which surgical procedures were divided into minor, moderate and extensive surgery and - likewise in our study - on the 1^st^ day after surgery serum FT3 levels decreased in all 3 groups, when compared to the baseline values. Serum FT4 levels did not change regardless of surgical procedure [14]. However, certain discrepancy exists between previous quoted results and our observations. In opposite to our findings, Murai et al. [14] demonstrated that serum TSH levels decreased significantly on 1^st^ day after surgery in the groups of moderate and extensive surgery.

It is worth stressing that in the our study patients who underwent minor surgery, mainly palliative due to unresectable abdominal tumours, were in the worse general condition before operation than patients who underwent major surgery. In our opinion, this fact - together with surgery extent - influenced the thyroid hormone metabolism and resulted in the similar final thyroid condition in both subgroups. This suggestion stays in accordance with results of earlier study in which authors demonstrated that the abnormalities in thyroid hormone metabolism were more frequent in patients admitted for urgent surgery than in patients scheduled for elective surgery. Furthermore, half out of the patients who underwent urgent surgery, persisted with changes in thyroid function tests in the late postoperative period, whereas most of the patients submitted to elective surgery presented an improvement in their thyroid function in the same period of time [6]. These results could indicate that the low T3 syndrome rather reflects the seriousness of patient’s general condition than the impact of surgery.

Our further statistical analyses revealed lower mean FT3 and FT4 concentrations after surgery in pancreatic tumours patients when compared to liver tumours patients. It is tempting to speculate that novel finding could be explained by an increased proportion of free thyroid hormones after liver surgery, caused by lower thyroid hormone binding proteins concentrations (hypoproteinemia) [15,16]. Another explanation might be based on an assumption that pancreatic surgery – generally - is a more serious procedure with more serious complications and lower free thyroid hormones levels are known to be a manifestation of particularly deep and serious metabolic disturbances.

Furthermore, it is worth mentioning that low T3 syndrome early after partial hepatectomy, characterized by increased activity of D3, associated with low serum and liver thyroid hormone levels, has been proved to have important role in the regenerating liver, in which a decrease in cellular T3 promotes an increase in proliferation [17]. The last cited report supports the hypothesis that low T3 syndrome is an adaptive process.

In order to make discussion on our results completed, we have to recall that although the extent of abdominal surgery does not impact significantly on the thyroid function, patients after pancreatic surgery are more likely to reveal lower FT4 levels.

## Competing interests

The authors declare that they have no competing interests.

## Authors’ contributions

KW-D participated in a design and coordination of the study, drafted the manuscript. AZ participated in coordination of the study. AD performed surgical treatment. JS performed surgical treatment. AL senior author, designed and coordinated the study, revised the text of manuscript. All authors have read and approved the final manuscript.
